# Computational Design of the Electronic Response for Volatile Organic Compounds Interacting with Doped Graphene Substrates

**DOI:** 10.3390/nano14221778

**Published:** 2024-11-05

**Authors:** Li Chen, David Bodesheim, Ahmad Ranjbar, Arezoo Dianat, Robert Biele, Rafael Gutierrez, Mohammad Khazaei, Gianaurelio Cuniberti

**Affiliations:** 1 Institute for Materials Science and Max Bergmann Center for Biomaterials, TUD Dresden University of Technology, 01062 Dresden, Germany; li.chen2@tu-dresden.de (L.C.); david.bodesheim@tu-dresden.de (D.B.); ahmad.ranjbar@tu-dresden.de (A.R.); arezoo.dianat@tu-dresden.de (A.D.); robert.biele@tu-dresden.de (R.B.); 2 Department of Physics, University of Tehran, Tehran 14395-547, Iran; mohammad.khazaei@ut.ac.ir; 3 Dresden Center for Computational Materials Science (DCMS), TUD Dresden University of Technology, 01062 Dresden, Germany

**Keywords:** work function change, surface dipole moment, N-doped graphene, VOCs, adsorption, DFT

## Abstract

Changes in the work function provide a fingerprint to characterize analyte binding in charge transfer-based sensor devices. Hence, a rational sensor design requires a fundamental understanding of the microscopic factors controlling the modification of the work function. In the current investigation, we address the mechanisms behind the work function change (WFC) for the adsorption of four common volatile organic compounds (toluene, ethanol, 2-Furfurylthiol, and guaiacol) on different nitrogen-doped graphene-based 2D materials using density functional theory. We show that competition between the surface dipole moment change induced by spatial charge redistribution, the one induced by the pure adsorbate, and the one caused by the surface deformation can quantitatively predict the work function change. Furthermore, we also show this competition can explain the non-growing work function change behavior in the increasing concentrations of nitrogen-doped graphenes. Finally, we propose possible design principles for WFC of VOCs interacting with N-doped graphene materials.

## 1. Introduction

Gas sensing technologies are critical for tracking environmental contaminants, maintaining safety in industrial settings, and protecting public health [1]. Volatile organic compounds (VOCs) represent a key target for these technologies, given their pervasive nature and hazards. VOCs are emitted from a variety of sources, including industrial activities, consumer products, and vehicle emissions, and their long-term exposure is linked to severe health and environmental consequences, such as respiratory disorders, damage to organs, and cancer [2,3].

The performance of gas sensors for detecting volatile organic compounds (VOCs) is primarily determined by the materials used in their design. Advanced materials, particularly state-of-the-art 2D materials, are of great interest for enhancing both the sensitivity, which allows for detection of low VOC concentrations, and the selectivity, which enables differentiation among various VOC types [4,5].

An important factor in evaluating the performance of these sensors is the work function (WF) ϕ (specifically for WF-based sensors), which characterizes the interaction between the sensor material and the target VOC molecule. The work function refers to the minimum energy needed for an electron to escape from the surface of a material, and the change in work function (WFC) Δϕ before and after gas molecule adsorption is widely measured as an important indicator or even direct electronic response for sensors (e.g., Schottky sensors) [6,7,8,9,10,11,12]. Computational methods such as density functional theory (DFT) are effective methods for analyzing the WFC of 2D materials upon the adsorption of VOC molecules. Numerous computational studies have been carried out to evaluate the potential sensing performance by WFC Δϕ of a variety of materials upon adsorption of VOCs [13,14,15,16,17,18,19,20]. However, despite the observed WFC values reported in these studies, there is a significant lack of understanding regarding the underlying mechanisms driving these changes.

To interpret the origin of the work function change, Leung et al. [21] developed the surface dipole moment model and revealed the work function change mechanism for single-atom adsorption on W surfaces. Subsequently this model has been applied to many systems such as single-atom adsorption [22], surface functionalization [23], and interfaces [24,25,26,27] to gain insights into the property/structure–property relationships for their systems of interest. Herein, we validate a model for physisorption of VOCs on typical 2D sensing materials. This facilitates a better rational design of sensing materials for WF-based sensors.

**Figure 1:**
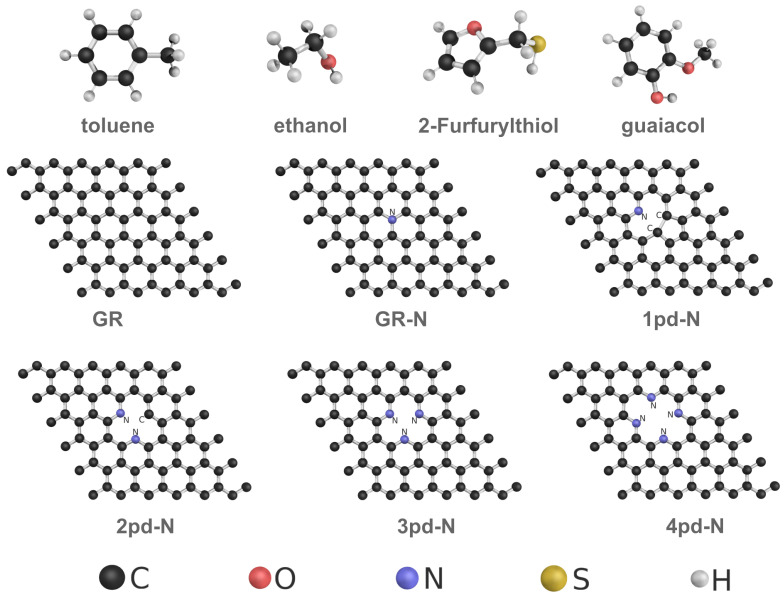
Ball-and-stick representation of VOC molecules and graphene-based substrates.

In this work, we investigate the WFC as an electronic response on different 2D N-doped graphenes upon adsorption of four common VOC molecules (toluene [28], ethanol [28], 2-Furfurylthiol [29], and guaiacol [29]). The substrates encompass pristine graphene (GR) and graphene substrates doped by single graphitic-N (GR-N) and pyridinic-N (1pd-N), as shown in Figure 1. Pyrrolic-N (pr-N) has been excluded, as it is not stable and converts to 1pd-N in all simulations. Furthermore, to probe the influence of local nitrogen and point defect concentration, we expanded from 1pd-N up to 4pd-N, expecting to increase the WF value [30,31]. Next, we validated the linear relationship of the WFC Δϕ and the surface dipole moment change $\Delta P_{\mathrm{tot}}$, and the observed behaviors of the WFC were quantitatively analyzed based on the components of the surface dipole moment change. Finally, we propose possible design principles for the WFC of VOCs by sensing for 2D materials.

## 2. Computational Methods

The Quantum ESPRESSO package [32,33,34] (version 7.0) was used to perform periodic DFT calculations with the Perdew–Burke–Ernzerhof (PBE) version of the generalized gradient approximation (GGA) exchange–correlation functional [35]. DFT-D3 was involved in all simulations to describe the van der Waals (vdW) interactions [36]. The cutoff energy of planar basis sets generated by the projector augmented wave (PAW) method was set to 60 Ry, while the charge density cut-off was 450 Ry. The Gaussian smearing spreading was employed with a width of 0.01 Ry. A (6 × 6) hexagonal supercell composed of 72 carbon atoms was modeled with a fixed vacuum thickness of 30.68 Å, ensuring negligible interaction of periodic images of isolated gas molecules. The atomic positions and lattice parameters (at the fixed z length) were optimized by constraining the cell length along the c direction to be constant until the total energies and the imposed force on the atoms became less than 10^−6^ Ry/cell and 10^−4^ Ry·Bohr^−1^, with <0.01 kbar of residual stress. A (2 × 2 × 1) Monkhorst–Pack grid was sampled in the first Brillouin zone for the k-points meshes. In addition, the coulomb interaction in the z direction was truncated for the structures’ periodic in the *x*–*y* plane [37] in all simulations to ensure constant vacuum energy and avoid heavy simulations caused by large vacuum layer lengths.

The charge transfer *Q* between the substrate and adsorbate was obtained using the Bader algorithm [38]. The sign of the charge transfer *Q* is defined as positive when the charges transfer from the substrate to the VOC molecules, and vice versa. The work function is defined as follows:

(1) $$\phi =E_{V}-E_{F},$$

where $E_{V}$ and $E_{F}$ are the vacuum energy and Fermi level, respectively.

Furthermore, a surface dipole moment analysis was carried out to study the behavior of the Δϕ. Leung et al. [21] derived the relation between Δϕ and the total surface dipole moment change $\Delta P_{\mathrm{tot}}$ as follows:

(2) $$\Delta \phi =-e/\varepsilon_{0}\;\cdot \;\left(V\;\cdot \;\overset{˚}{A}\right)\;\cdot \;\Delta P_{\mathrm{tot}}\;\cdot \;\left(e/\overset{˚}{A}\right),$$

where *e* and $\varepsilon_{0}$ are the unit charge and vacuum permittivity, respectively. Leung et al. [21] and Khazaei et al. [23] further divided $\Delta P_{\mathrm{tot}}$ into three components: (a) the surface dipole moment change induced by spatial charge redistribution $\Delta p_{\mathrm{cplx}}$, (b) the surface dipole moment induced by the pure adsorbate $p_{a}$, and (c) the surface dipole moment induced by surface deformation after and before adsorption ($p_{s}-p_{0}$). Then, Equation (2) turns into the following equation:

(3) $$\Delta \phi =-e/\varepsilon_{0}\;\cdot \;\left(V\;\cdot \;\overset{˚}{A}\right)\;\cdot \;\left(\Delta p_{\mathrm{cplx}}+p_{a}+p_{s}-p_{0}\right)\;\cdot \;\left(e/\overset{˚}{A}\right).$$

Each dipole moment component as a function of the *z* direction was calculated using the *x*–*y* planar average of the corresponding charge densities via the integral $p\left(z\right)=\int_{z_{0}}^{z}zn\left(z\right)dz$, where $z_{0}$ is set at the bottom of the cell. For the electron charge transfer, we have the following relationship [21]:

(4) $$\Delta n\left(z\right)=n_{\mathrm{ads}-\mathrm{sub}}\left(z\right)-\left[n_{s}\left(z\right)+n_{a}\left(z\right)\right],$$

where Δn(z) is the charge density redistribution, $n_{\mathrm{ads}-\mathrm{sub}}\left(z\right)$ is the total charge density of the adsorbate–substrate complex system, and $n_{s}\left(z\right)$/$n_{a}\left(z\right)$ are the charge density for the isolated substrate/adsorbate computed by removing one component from the complex system without further optimization. Therefore, the $\Delta p_{\mathrm{cplx}}$ component for the charge redistribution term is then calculated using Δn(z). The total surface dipole moment change $\Delta P_{\mathrm{tot}}$ is defined as the total surface dipole moment difference between the adsorbate–substrate system and the isolated substrate (before adsorption), which has a corresponding total charge density of $n_{0}\left(z\right)$. Hence $n_{\mathrm{ads}-\mathrm{sub}}\left(z\right)$ and $n_{0}\left(z\right)$ in Equation (4) would be utilized for the $\Delta P_{\mathrm{tot}}$ calculation.

The resulting values of $p_{s}$ − $p_{0}$, $p_{a}$, and $\Delta p_{\mathrm{cplx}}$ were normal to the substrate’s *x*–*y* plane, where a positive value indicates a dipole pointing out to the vacuum, and vice versa. Their corresponding values were obtained at $z=30\;(\overset{˚}{A})$, where these surface dipole moment components remained constant with increasing *z* length.

## 3. Results and Discussion

### 3.1. Physisorption Interaction Type

In order to find the most favorable configurations, we considered 7 to 12 different initial configurations for each adsorption case, including vertical, horizontal, and intermediate orientations of molecules and distinct adsorption sites. The configurations with the lowest adsorption energy $E_{\mathrm{ads}}$ were selected for further electronic property calculations. The $E_{\mathrm{ads}}$ is defined as $E_{\mathrm{ads}}=E_{\mathrm{cplx}}-E_{s}-E_{a}$, where $E_{\mathrm{cplx}}$ is the total energy of the optimized adsorbate–substrate complex system, while $E_{s}$ and $E_{a}$ are the total energies of the isolated substrate and gas molecule, respectively. The adsorption energies for all initial configurations are depicted in Table S1.

The vertical distance between the lowest atom of the VOC molecules (always the H atom in our case) and the substrate plane (since there is no dramatic deformation of the substrate upon adsorption, as shown in Figures S1–S4) for these low-energy configurations are listed in Table 1. As shown in Table 1, all of the vertical distances are longer than typical N-H and C-H covalent bonds, such as ∼1.1 Å in methylamine [39,40,41], indicating the physisorption interaction type for our systems.

### 3.2. Work Function Change

The change in work function is calculated and used as a indicator for the substrate’s electronic response towards the VOC molecules. In principle, a large WFC upon adsorption implies a higher sensitivity of the substrate to adsorbate. Differences in the WFC between different molecules adsorbing on the same substrate provide information into the selectivity performance of this substrate towards these adsorbates.

**Figure 2:**
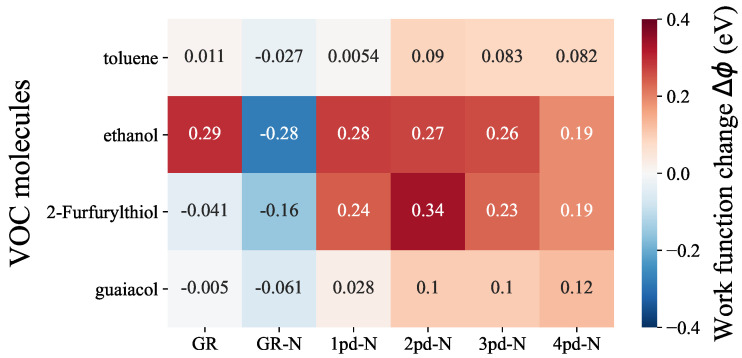
Heatmap of work function change Δϕ comparisons among different odorant–substrate combinations. The labeled number indicates the corresponding work function change value.

As depicted in Figure 2, within each molecule row, we can make comparisons regarding the sensitivity of each substrate to a specific VOC molecule, while within each column, the selectivity of a particular substrate towards various VOC molcules can be assessed. Upon examining the molecule rows, it becomes evident that toluene and guaiacol evoke rather smaller responses in all substrates, with Δϕ values consistently falling below 0.1 eV compared to the other two, even though, there is still a subtle increase in Δϕ values when transitioning from pristine graphene (GR) to N-doped graphene (GR-N) and multiple pd-N substrates. In contrast, ethanol and 2-Furfurylthiol exhibit overall higher responses compared to the other two odorants. In the ethanol row, the relatively large absolute values of Δϕ can be attributed to the high electronegativity of the oxygen atom of ethanol, resulting in substantial charge transfer. Importantly, the Δϕ values for ethanol are consistently large and nearly identical for all substrates, indicating that both graphene and N-doped graphene hold significant promise for the detection of ethanol. Regarding 2-Furfurylthiol adsorption, the richer variety of work function changes in this row suggests that the substrates demonstrate distinguishable responses. Notably, 2pd-N displays the most substantial Δϕ of 0.34 eV, whereas GR exhibits almost no sensitivity to 2-Furfurylthiol, yielding a Δϕ value of −0.041 eV. From a selectivity perspective (column-wise), GR-N and 2pd-N exhibit relatively superior discrimination capabilities towards ethanol and 2-Furfurylthiol. This is evident from the more pronounced differences in Δϕ values in these columns compared to the toluene and guaiacol. Although the introduction of nitrogen into the substrates generally raises the Δϕ values when compared to pure graphene, this does not necessarily enhance selectivity towards ethanol, 2-Furfurylthiol, toluene, or guaiacol. In fact, the stronger interaction between substrate and adsorbate, as reflected in the adsorption energy $E_{\mathrm{ads}}$ trend presented in Figure S5, was expected to lead to a stronger response, e.g., Δϕ. However, either sensitivity or selectivity appears to deteriorate, particularly for 3pd-N and 4pd-N substrates, which does not satisfy our expectation regarding the enhancement by adding multiple pyrodinic-nitrogen atoms and enlarging the point defect concentration, as mentioned in the introduction.

### 3.3. Charge Transfer vs. Work Function Change

In order to obtain insights into the behavior of work function changes observed in Figure 2, the total net charge transfer *Q* is compared with the change in work function, since *Q* on the substrate is tightly correlated to Δϕ. It is generally anticipated that the adsorption of molecules induces charge transfer as a result of the electronegativity difference between the adsorbate and adsorbent. Once the adsorbate donates electrons, the substrate likely needs less energy to spill out the electrons again; hence, the work function will decrease, leading to a negative Δϕ, and vice versa. As a consequence, the charge transfer *Q* is anticipated to correlate directly with the magnitude of the change in the work function Δϕ, implying that variations in Δϕ should be mirrored by corresponding variations in *Q*.

**Figure 3:**
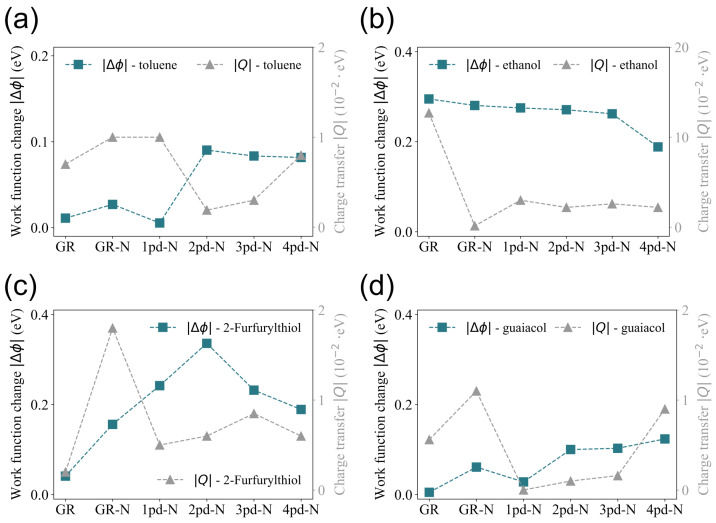
Comparison of trends in the absolute value of work function change Δϕ and charge transfer *Q* of the substrates towards the adsorption of (**a**) toluene, (**b**) ethanol, (**c**) 2-Furfurylthiol, and (**d**) guaiacol.

However, this comparison is presented in Figure 3, which depicts an inconsistent trend between the variations in Δϕ and *Q* across the different substrates (GR was taken as the reference point). This inconsistent correlation has also been observed in other studies [14]. For instance, while the change in work function and charge transfer for 2-Furfurylthiol on the GR, 1pd-N, 2pd-N, and 4pd-N substrates aligns with the above-mentioned expectations, discrepancies are observed for the GR-N and 3pd-N substrates. These anomalies suggest that the relationship between Δϕ and *Q* is not universally correlated and might be influenced by additional factors.

### 3.4. Surface Dipole Moment Change Validation

To further investigate the mechanisms underlying the observed Δϕ behavior for N-doped graphene substrates, we carried out an analysis of surface dipole moment changes.

**Figure 4:**
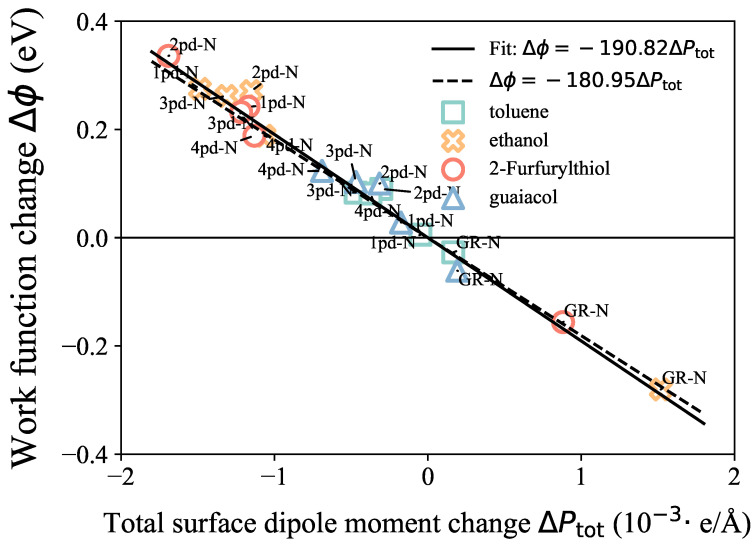
Linear correlation between work function change Δϕ (*y*-axis) and surface dipole moment change $\Delta P_{\mathrm{tot}}$ (*x*-axis). The straight solid and dashed lines present the fitted and referred linear correlation, respectively.

First, to validate the model on our systems, the linear correlation between the work function change Δϕ and the surface dipole moment change $\Delta P_{\mathrm{tot}}$ is presented in Figure 4. The $\Delta P_{\mathrm{tot}}$ values were calculated by considering the difference in the total dipole moment between the adsorbate–substrate system and the isolated substrate, specifically $\Delta P_{\mathrm{tot}}=p_{\mathrm{tot}}-p_{0}$. The proportionality coefficient obtained from our fitting result is −190.82 $V\;\cdot \;\overset{˚}{A}$, as shown in Equation (2), which is in good agreement with the constant value of $-e/\varepsilon_{0}=-180.95\;\left(V\;\cdot \;\overset{˚}{A}\right)$. These results indicate that this surface potential model is not only valid for chemisorbed systems of bulk, as Leung et al. [21] and Khazaei [23] reported, but is also applicable to the physisorbed 2D systems in this work due to the negligible shifts in the Fermi level for both kinds of systems. Based on this linear relationship, the trend of $\Delta P_{\mathrm{tot}}$ could approximate the trend of the work function change Δϕ, and we could also obtain insights into the possible reasons for the Δϕ trend through the $\Delta P_{\mathrm{tot}}$ components’ competition.

### 3.5. Surface Dipole Moment Change Decomposition

**Figure 5:**
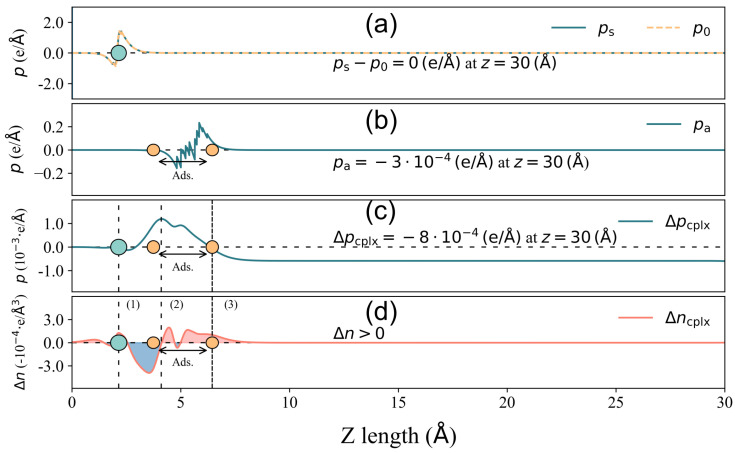
Total surface dipole moment change decomposition for 2-Furfurylthiol adsorbed on 4pd-N substrate. (**a**) Components of surface deformation $p_{s}$ − $p_{0}$. (**b**) Surface dipole moment resulting from the deformation of the VOC molecule upon adsorption $p_{a}$. (**c**) $\Delta p_{\mathrm{cplx}}$ owing to the spatial charge redistribution in the complex adsorbate–adsorbent system. (**d**) Charge density difference distribution Δn of the *x*–*y* planar average along the *z* direction. The grey circle is the location of the substrate, and the area between the two orange circles is the adsorbate location, where the lowest and highest atoms of the VOC molecule are denoted by the two atoms. In (**c**,**d**), the curve is divided into several regions: (1), (3) blue areas indicating electron depletion and (2) red area indicating electron accumulation. The dense dashed line denotes the highest position for $\Delta p_{\mathrm{cplx}}=0$, and the sign of Δn is labeled in (**d**).

**Figure 6:**
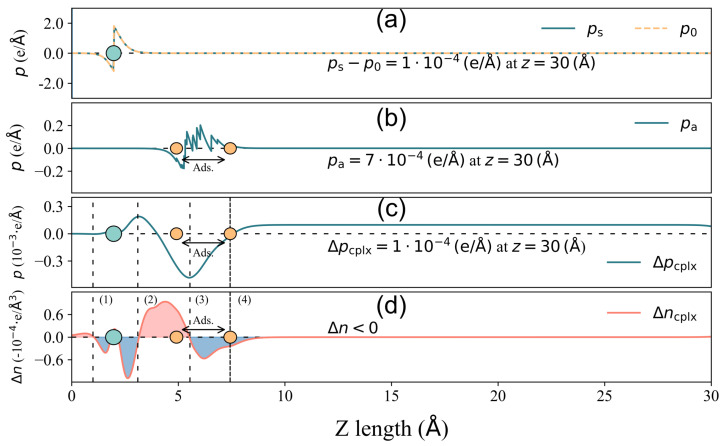
The same as Figure 5, but for GR-N.

Now, to further investigate the correlation behavior between *Q* and Δϕ in Figure 3, we take 2-Furfurylthiol adsorbed on GR-N and 4pd-N as typical examples, which have Δϕ and *Q* pairs of −0.156 eV, 0.018 e and 0.189 eV, −0.006 e, respectively. Their corresponding descriptions of components $\Delta p_{\mathrm{cplx}}$, $p_{a}$, and $p_{s}-p_{0}$ for total surface dipole moment change $\Delta P_{\mathrm{tot}}$ decomposition are displayed in Figure 5 and Figure 6.

In the case of 4pd-N, as depicted in Figure 5, the electron accumulation/depletion in regions (1) and (3)/(2) results in a tiny total net charge transfer *Q* of $6\times 10^{-4}$ e from the substrate to the odorant. The charge density changes in regions (1) and (2) effectively compensate for each other, leading to a zero value of $\Delta p_{\mathrm{cplx}}$ until the onset of region (3) in panel (c). The charge redistribution in the outer layer of region (3) contributes significantly to the final value of $\Delta p_{\mathrm{cplx}}$, amounting to $8\times 10^{-4}\;e/\overset{˚}{A}$, which is considerably larger than the other two components ($p_{a}=-3\times 10^{-4}\;\left(e/\overset{˚}{A}\right)$ and $p_{s}-p_{0}\approx 0\;\left(e/\overset{˚}{A}\right)$). Hence, in this case, it is the charge redistribution mechanism that primarily drives the surface dipole moment change under a small *Q*. In the GR-N case, as illustrated in Figure 6, electrons accumulate/deplete in regions (2)/(1), (3), and (4). Similar to the 4pd-N case, charge redistribution (depletion) within the outer layer of the adsorbate in region (4) leads to $\Delta p_{\mathrm{cplx}}=1\times 10^{-4}\;\left(e/\overset{˚}{A}\right)$, pointing out to the vacuum. However, the spatial charge redistribution results in a greater net charge transfer *Q* of 0.018e from the adsorbate to the adsorbent, which is three times higher than the *Q* observed in the 4pd-N case. Nonetheless, the pure adsorbate-induced dipole moment, $p_{a}=7\times 10^{-4}\;\left(e/\overset{˚}{A}\right)$, dominates the $\Delta P_{\mathrm{tot}}$ change in comparison to $\Delta p_{\mathrm{cplx}}=1\times 10^{-4}\;\left(e/\overset{˚}{A}\right)$.

From both cases, it is shown that the total net charge transfer *Q* does not necessarily correlate with the total surface dipole moment change $\Delta P_{\mathrm{tot}}$ (and hence the work function change Δϕ).

**Figure 7:**
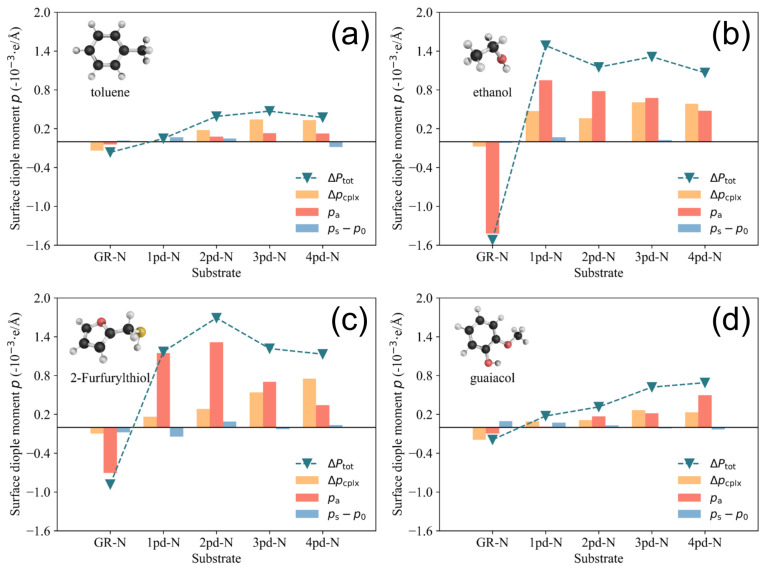
Histograms of total surface dipole moment change decomposition for (**a**) toluene, (**b**) ethanol, (**c**) 2-Furfurylthiol, and (**d**) guaiacol. The variation in the negative total surface dipole moment change $\Delta P_{\mathrm{tot}}$ and the distribution of the components $\Delta p_{\mathrm{cplx}}$, $p_{a}$, and $p_{s}-p_{0}$ are presented.

Furthermore, there is almost no contribution from $p_{s}-p_{0}$, indicating that the adsorption of 2-Furfurylthiol induces negligible substrate deformation. As for other cases, the mechanism of charge redistribution could be more complicated, but the competition between $\Delta p_{\mathrm{cplx}}$ and $p_{a}$ remains, which results in the inconsistent scaling trend between charge transfer and total surface dipole moment change $\Delta P_{\mathrm{tot}}$ (work function change Δϕ). In order to make an overall comparison of the surface dipole moment change between various cases, the value of $\Delta P_{\mathrm{tot}}$ and its corresponding components are presented in Figure 7. To make a direct comparison between $\Delta P_{\mathrm{tot}}$ and Δϕ, the minus sign is assigned to the unit in Figure 7.

As illustrated in Figure 7, the component $p_{s}-p_{0}$ (represented by the blue bar), which characterizes the surface dipole moment change induced by surface deformation, is not as sensitive as the other two components, which could typically yield large-value increments by changing the substrates. It is worth noting that the positive $\Delta p_{\mathrm{cplx}}$ and $\Delta p_{a}$ components observed in the GR-N columns in all four subfigures lead to a positive $\Delta P_{\mathrm{tot}}$ (and hence negative Δϕ in Figure 2) upon the adsorption of the odorants. This is in contrast to other pyridinic-N-doped substrates, as discussed in Figure 5, which yield negative values for these surface dipole moment components and finally result in a positive Δϕ. A notable general trend is the continuous increase in $\Delta p_{\mathrm{cplx}}$ (depicted by the orange bar) as a function of the number of pyridinic-N atoms for the four odorants. This phenomenon can be attributed to the growing number of pyridinic-N atoms, which introduces additional dangling bonds due to the presence of an unpaired electron localized at these pyridinic-N sites [42]. These dangling bonds facilitate substantial charge rearrangement, resulting in the amplification of $\Delta p_{\mathrm{cplx}}$. However, it is important to note that the increasing $\Delta p_{\mathrm{cplx}}$ value does not always lead to a monotonic increase in the change of the total surface dipole moment, $\Delta P_{\mathrm{tot}}$. In the cases of 2-Furfurylthiol and ethanol adsorption on 3pd-N and 4pd-N substrates, as shown in (c) and (d) of Figure 7, the $p_{a}$ components decrease, while $\Delta p_{\mathrm{cplx}}$ increases. This phenomenon might explain why the work function change Δϕ for the adsorption of 2-Furfurylthiol and ethanol on the 3pd-N and 4pd-N substrates does not exhibit a continuous increase as more pyridinic-N atoms are added.

In Figure 2, we have already seen the Δϕ difference for toluene/guaiacol and 2-Furfuryl-thiol/ethanol. As for toluene, it is a weakly polarized molecule with the smallest intrinsic dipole moment; therefore, the charge rearrangements resulted in $\Delta p_{\mathrm{cplx}}$ varying only within a limited range despite the increasing enhancement of nitrogen doping. Similarly, even though guaiacol has the largest in-plane intrinsic dipole moment among the four molecules, it contributes only to a limited extent to the spatial charge redistribution, as the phenyl ring of guaiacol, as well as toluene, is positioned horizontally on the substrates to maximize the van der Waals interaction and minimize the adsorption energy $E_{\mathrm{ads}}$.

Additionally, the high electronegative oxygen atoms of guaiacol do not contribute to the charge redistribution. The reason for this could be that the phenol hydrogen atom and oxygen atom adjacent to the methyl group already construct a robust intramolecular hydrogen bond [43], which leads to a weaker interaction with the substrate. Therefore, though the sensitivity of guaiacol is slightly greater than that of toluene due to the increase in the $p_{a}$ component, their tiny $\Delta p_{\mathrm{cplx}}$ and $p_{a}$ values limit the corresponding $\Delta P_{\mathrm{tot}}$ (hence Δϕ) within a small range. In contrast, there is a significantly larger increase in $\Delta p_{\mathrm{cplx}}$ and $p_{a}$ for the larger polarized 2-Furfurylthiol and ethanol compared to toluene and guaiacol, which could be attributed to the presence of strong polar functional groups on the odorants such as S and −OH. However, for these VOCs molcules, as shown in (b) and (c) in Figure 7, there is a drop in the $p_{a}$ component as the pyridinic-N atoms number increases, especially on 3pd-N and 4pd-N substrates. Even though the $\Delta p_{\mathrm{cplx}}$ remains increasing, the $\Delta P_{\mathrm{tot}}$ (hence the Δϕ) becomes unchanged due to the decline in $p_{a}$. This might explain why the selectivity ultimately deteriorates while continuing to induce the pyrodinic-N atoms into substrates, as reflected in the work function change shown in Figure 2.

### 3.6. Design Principle of Δϕ for VOC Sensing

By validating the linear relationship between $\Delta P_{tot}$ and Δϕ and understanding the underlying mechanism, we propose possible design principles of sensing materials and VOC pairs with their expected Δϕ values.

- $\Delta p_{\mathrm{cplx}}$: One could induce multiple highly electronegative atoms into the adsorption site on the substrate, since it will bring more dangling bonds and yield charge redistribution, and vice versa, as reflected in the $\Delta p_{\mathrm{rcplx}}$ values presented in Figure 7.
- $p_{a}$: While the out-of-plane dipole moment of VOC molecules can be tuned, adjusting the sign of $p_{a}$ by substituting different types of dopant atoms (e.g., pyridinic-N and pyrrolic-N) could further enhance the directional contrast of the Δϕ difference.
- $p_{s}-p_{0}$: Increasing the $p_{s}-p_{0}$ value is not recommended, even though this term also has a large influence on the Δϕ value, as large deformation of the substrate is detrimental to the stability and durability of sensing materials.

## 4. Conclusions

In this work, the work function change (WFC) is calculated as a electronic response for four VOC molcules—toluene, ethanol, 2-Furfurylthiol, and guaiacol—physisorbed onto pristine and N-doped graphene substrates using DFT. 2-Furfurylthiol and ethanol induced a significantly larger WFC than toluene and guaiacol. Among the N-doped substrates, GR-N and 2pd-N exhibited good discrimination toward sensing 2-Furfurylthiol and ethanol. However, the sensitivity was not further enhanced by introducing more pyridinic-N atoms into the substrate, especially for 3pd-N and 4pd-N. To explain this, the surface dipole moment analysis for the 2-Furfurylthiol and ethanol cases showed that the charge redistribution scale (characterized by the $\Delta p_{\mathrm{cplx}}$) increases, while the induced adsorbate dipole ($p_{a}$) drops with respect to the increasing number of pyridinic-N atoms. This gives rise to the non-increasing sensitivity of 2-Furfurylthiol and ethanol on the 3pd-N and 4pd-N substrates. This shows that the competition between the surface dipole moment components leads to the work function change behavior on our N-doped graphene samples. Finally, based on this knowledge, we proposed possible design principles of WFC for VOCs interacting with N-doped graphene by tuning the surface dipole moment components’ values. These computational findings suggest a new way to investigate the work function change behavior for 2D materials and hence offer principles for the rational design of materials and their responses to VOCs.

## Figures and Tables

**Table 1:** Vertical distance between the lowest atom on the VOC molecule along the slab direction and the substrate plane. Distances are in Å.

	GR	GR-N	1pd-N	2pd-N	3pd-N	4pd-N
Toluene	2.90	3.01	2.77	2.36	2.13	1.81
Ethanol	3.40	2.95	2.86	2.77	1.68	1.29
2-Furfurylthiol	2.60	3.14	2.41	2.09	1.89	1.57
Guaiacol	2.58	2.60	2.46	2.32	2.16	1.80

## Data Availability

The data that support the findings of this study are available from the corresponding author upon reasonable request.

## Supplementary Materials

The following supporting information can be downloaded at: https://www.mdpi.com/article/10.3390/nano14221778/s1, Table S1: Adsorption energy $E_{\mathrm{ads}}$ for all adsorption cases. The lowest $E_{\mathrm{ads}}$ is highlighted in bold. The adsorption on graphene serves as a reference case. Therefore, only four initial configurations were taken into account. Figure S1: The most energy-favorable adsorption configurations for toluene adsorption. Figure S2: The most energy-favorable adsorption configurations for ethanol adsorption. Figure S3: The most energy-favorable adsorption configurations for 2-Furfurylthiol adsorption. Figure S4: The most energy-favorable adsorption configurations for guaiacol adsorption. Table S2: Adsorption energy $E_{\mathrm{ad}}$ and Charge transfer *Q* comparison for the odor molecules with the most stable configurations over pristine graphene, N-gra, 1pd-N, 2pd-N, 3pd-N, and 4pd-N substrates. Positive values of charge transfer indicate electron transfer from the molecule to the substrate, while negative values indicate electron transfer from the substrate to the molecule. Figure S5: Adsorption energy comparison for the odorants adsorption on different substrates is shown.
