# Supplementary material for: Computational Design of the Electronic Response for Volatile Organic Compounds Interacting with Doped Graphene Substrates
Source: Nanomaterials (Basel). 2024 Nov 5;14(22):1778. doi: 10.3390/nano14221778 (PMC11597515; doi:10.3390/nano14221778)
Supplement: Supplementary file 1 [file nanomaterials-14-01778-s001.zip › nanomaterials-3247945-supplementary.pdf]

SUPPORTING INFORMATION For Computational Design of the Electronic Response for Volatile Organic Compounds Interacting with Doped Graphene Substrates

Li Chen[1], David Bodesheim[1], Ahmad Ranjbar[1], Arezoo Dianat[1], Robert Biele[1], Rafael Gutierrez*[1], Mohammad Khazaei[2], and Gianaurelio Cuniberti*[1,3]

[1]*Institute for Materials Science and Max Bergmann Center for Biomaterials, TUD Dresden University of Technology, 01062 Dresden, Germany*

[2]*Department of Physics, University of Tehran, 14395-547 Tehran, Iran*

[3]*Dresden Center for Computational Materials Science (DCMS), TUD Dresden University of Technology, 01062 Dresden, Germany*

October 22, 2024

*Corresponding author: rafael.gutierrez@tu-dresden.de (R.G.); gianaurelio.cuniberti@tu-dresden.de (G.C.)

S1 Collection of adsorption energy of initial configurations

In order to find the most favorable configurations, we considered 7 to 12 different initial configurations as starting points for each adsorption case including vertical, horizontal, and intermediate orientation of molecules and distinct adsorption sites. The configurations with the lowest adsorption energy E_{ads} were selected out and used to calculate further electronic properties.

Substrate	Configuration number	toluene	ethanol	2-Furfurylthiol	guaiacol
	1	-0.197	-0.159	-0.355	-0.311
Pristine graphene (GR)	2	-0.367	-0.204	-0.395	-0.357
	3	-0.373	-0.248	-0.320	-0.373
	4	**-0.375**	**-0.282**	**-0.325**	**-0.559**
	1	-0.503	-0.285	-0.329	-0.616
	2	**-0.506**	-0.274	-0.330	-0.601
	3	-0.506	-0.226	**-0.36**	-0.607
Single graphitic-N doped graphene (GR-N)	4	-0.255	-0.150	-0.271	-0.523
	5	-0.373	-0.236	-0.372	-0.568
	6	-0.501	-0.285	-0.396	-0.603
	7	-0.33	**-0.294**	-0.399	-0.605
	8	-0.338	-0.209		
	1	-0.36	-0.33	-0.302	-0.3837
	2	-0.324	**-0.380**	-0.364	-0.507
	3	-0.317	-0.377	-0.378	-0.356
	4	-0.383	-0.227	-0.319	**-0.613**
	5	-0.369	-0.352	-0.389	-0.545
Single pyridinic-N doped graphene (1pd-N)	6	-0.369	-0.352	-0.389	-0.545
	7	-0.392	-0.276	-0.373	-0.509
	8	**-0.399**	-0.379	-0.327	-0.594
	9	-0.376		-0.303	
	10			-0.314	
	11			-0.38	
	12			**-0.39**	
	1	-0.321	-0.316	-0.348	-0.387
	2	-0.340	**-0.319**	**-0.379**	-0.507
	3	-0.266	-0.307	-0.365	-0.301
	4	-0.33	-0.222	-0.325	**-0.611**
two pyridinic-N doped graphene (2pd-N)	5	-0.341	-0.161	-0.356	-0.544
	6	-0.374	-0.244	-0.35	-0.535

continued from previous page

continued from previous page

Substrate	Configuration number	toluene	ethanol	2-Furfurylthiol	guaiacol
	7	**-0.386**	-0.3146	-0.360	-0.596
	8	-0.30	-0.398	-0.382	-0.541
	9		-0.318		
	10		-0.379		
	1	-0.316	-0.537	**-0.533**	-0.319
	2	-0.385	-0.536	-0.32	-0.660
	3	-0.318	**-0.540**	-0.348	-0.343
	4	**-0.527**	-0.533	-0.381	-0.660
	5	-0.346	-0.265	-0.526	-0.539
three pyridinic-N doped graphene (3pd-N)	6	-0.36	-0.539	-0.527	-0.544
	7	-0.342	-0.514	-0.377	-0.660
	8	-0.363	-0.260	-0.389	**-0.661**
	9	-0.326	-0.270		-0.657
	10	-0.365	-0.311		
	11		-0.34		
	12		-0.265		
	1	-0.590	-0.557	-0.652	**-0.732**
	2	-0.591	-0.381	-0.558	-0.731
	3	-0.34	-0.562	-0.618	-0.682
	4	-0.550	-0.559	-0.603	-0.684
	5	-0.501	-0.266	-0.612	-0.534
four pyridinic-N doped graphene (4pd-N)	6	-0.566	-0.307	-0.378	-0.538
	7	-0.566	-0.554	-0.568	-0.620
	8	-0.592	-0.561	-0.373	-0.627
	9	-0.550	-0.37	-0.593	-0.703
	10		**-0.572**	-0.681	
	11			**-0.698**	

Table S1: Adsorption energy E_{ads} for all adsorption cases. The lowest E_{ads} is highlighted in bold. The adsorption on graphene serves as a reference case. Therefore, only four initial configurations were taken into account.

S2 The structures of the most energy-favorable configurations

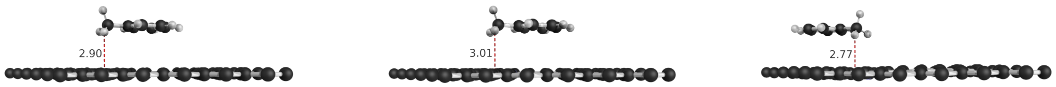

(a) Left to right: front view for toluene adsorption on GR, GR-N, and 1pd-N.

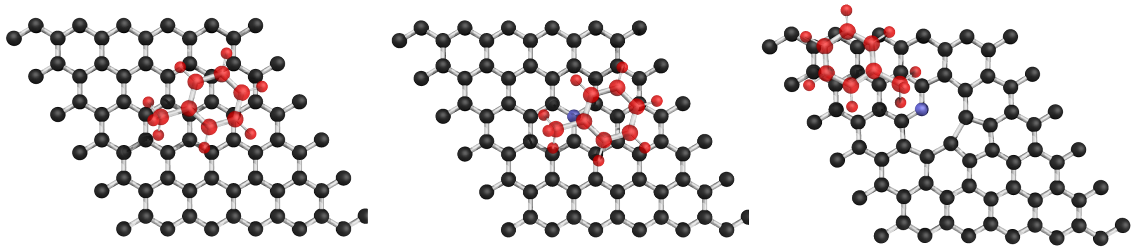

(b) Left to right: top view for toluene adsorption on GR, GR-N, and 1pd-N.

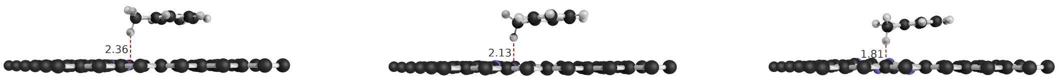

(c) Left to right: front view for toluene adsotption on 2pd-N, 3pd-N, and 4pd-N.

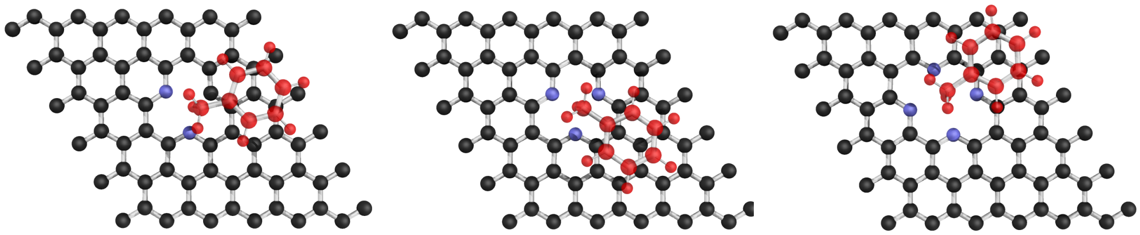

(d) Left to right: top view for toluene adsotption on 2pd-N, 3pd-N, and 4pd-N.

Figure S1: The most energy-favorable adsorption configurations for toluene adsorption.

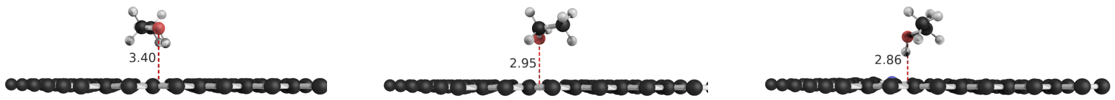

(a) Left to right: front view for ethanol adsorption on GR, GR-N, and 1pd-N.

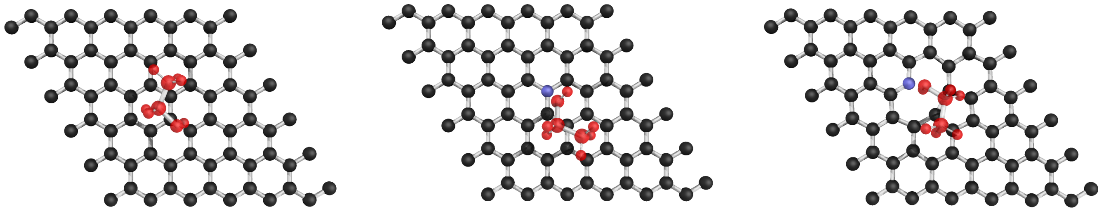

(b) Left to right: top view for ethanol adsorption on GR, GR-N, and 1pd-N.

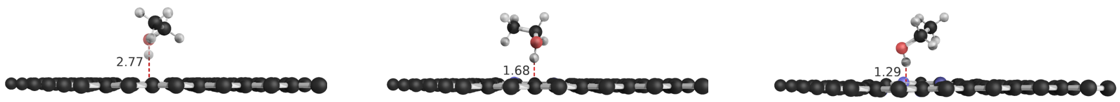

(c) Left to right: front view for ethanol adsorption on 2pd-N, 3pd-N, and 4pd-N.

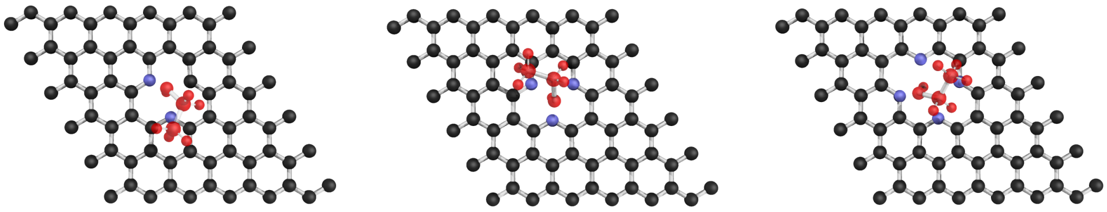

(d) Left to right: top view for ethanol adsorption on 2pd-N, 3pd-N, and 4pd-N.

Figure S2: The most energy-favorable adsorption configurations for ethanol adsorption.

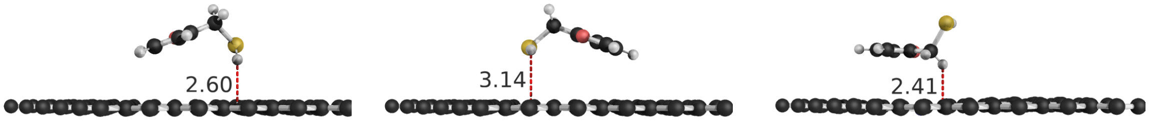

(a) Left to right: front view for 2-Furfurylthiol adsorption on GR, GR-N, and 1pd-N.

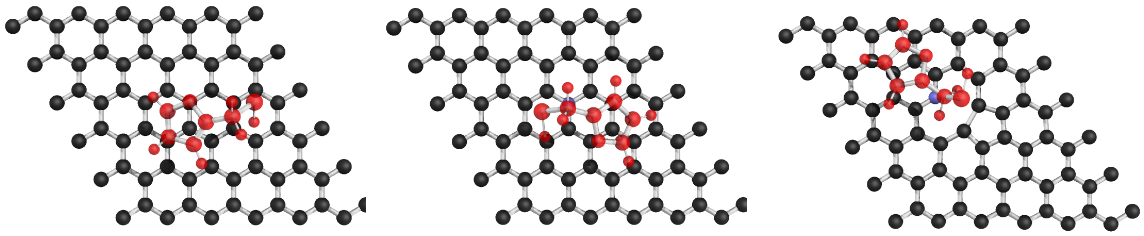

(b) Left to right: top view for 2-Furfurylthiol adsorption on GR, GR-N, and 1pd-N.

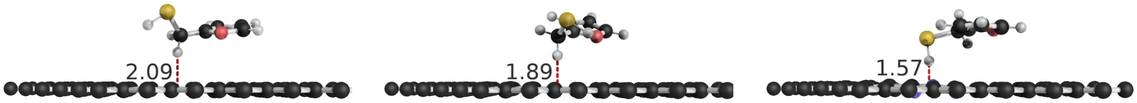

(c) Left to right: front view for 2-Furfurylthiol adsorption on 2pd-N, 3pd-N, and 4pd-N.

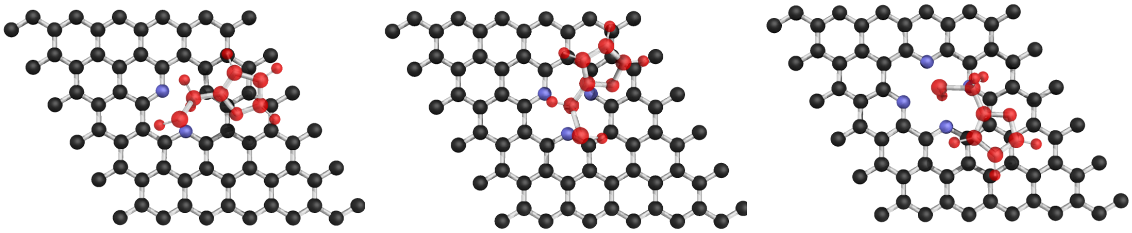

(d) Left to right: top view for 2-Furfurylthiol adsorption on 2pd-N, 3pd-N, and 4pd-N.

Figure S3: The most energy-favorable adsorption configurations for 2-Furfurylthiol adsorption.

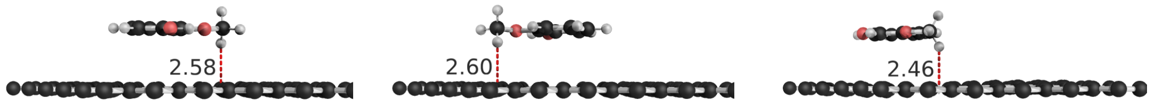

(a) Left to right: front view for guaiacol adsorption on GR, GR-N, and 1pd-N.

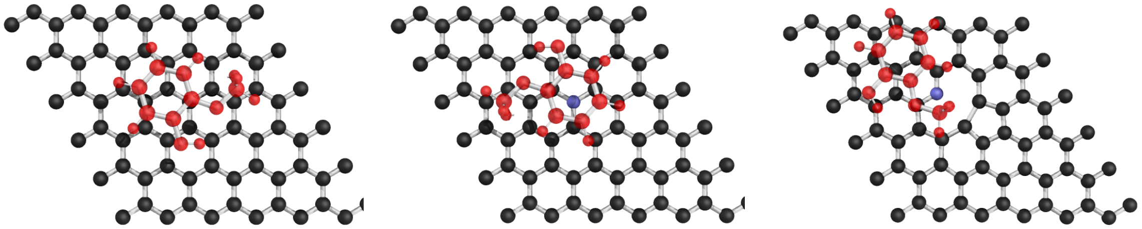

(b) Left to right: top view for guaiacol adsorption on GR, GR-N, and 1pd-N.

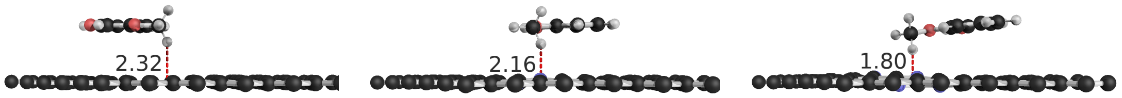

(c) Left to right: front view for guaiacol adsorption on 2pd-N, 3pd-N, and 4pd-N.

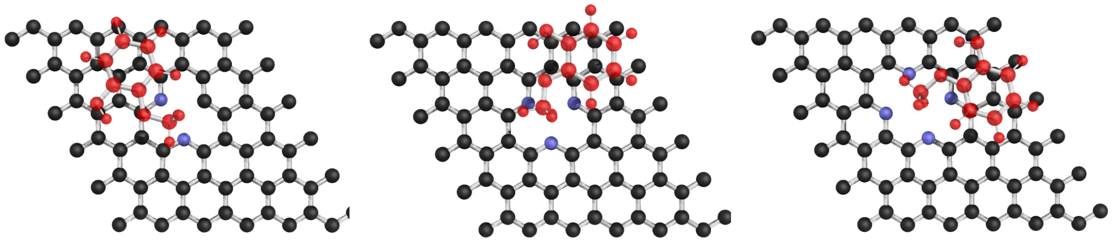

(d) Left to right: top view for guaiacol adsorption on 2pd-N, 3pd-N, and 4pd-N.

Figure S4: The most energy-favorable adsorption configurations for guaiacol adsorption.

S3 Electronic properties of the complex system

Table S2: Adsorption energy E_{ad} and Charge transfer Q comparison for the odor molecules with the most stable configurations over pristine graphene, N-gra, 1pd-N, 2pd-N, 3pd-N, and 4pd-N substrates. Positive values of charge transfer indicate electron transfer from the molecule to the substrate, while negative values indicate electron transfer from the substrate to the molecule.

	Substrate	toluene	ethanol	2-Furfurylthiol	guaiacol
Chemical formula		C_7H_8	C_2H_5OH	C_5H_6OS	$C_7H_8O_2$
Molecular mass (g/mol)		92.14	46.07	114.17	124.14
Dipole moment (Debye)		0.4297	1.6575	1.8376	2.6641
Adsorption energy E_{ads} (eV)	GR	-0.473	-0.282	-0.425	-0.559
	GR-N	-0.506	-0.294	-0.456	-0.616
	1pd-N	-0.499	-0.380	-0.459	-0.612
	2pd-N	-0.486	-0.419	-0.479	-0.610
	3pd-N	-0.527	-0.540	-0.533	-0.661
	4pd-N	-0.591	-0.572	-0.698	-0.732
Charge transfer Q (e)	GR	0.007	-0.127	0.002	0.0056
	GR-N	0.010	0.0015	0.018	0.011
	1pd-N	0.010	-0.03	0.005	0.00001
	2pd-N	0.0019	-0.022	-0.006	0.001
	3pd-N	0.003	-0.026	-0.0085	0.0016
	4pd-N	0.008	-0.022	-0.006	0.009
Work function change $\Delta\phi$ (eV)	GR	0.011	0.295	-0.041	-0.005
	GR-N	-0.027	-0.2805	-0.156	-0.061
	1pd-N	0.0054	0.275	-0.242	0.028
	2pd-N	0.0902	0.271	0.336	0.1
	3pd-N	0.0834	0.262	0.232	0.1028
	4pd-N	0.0816	0.1885	0.189	0.1235

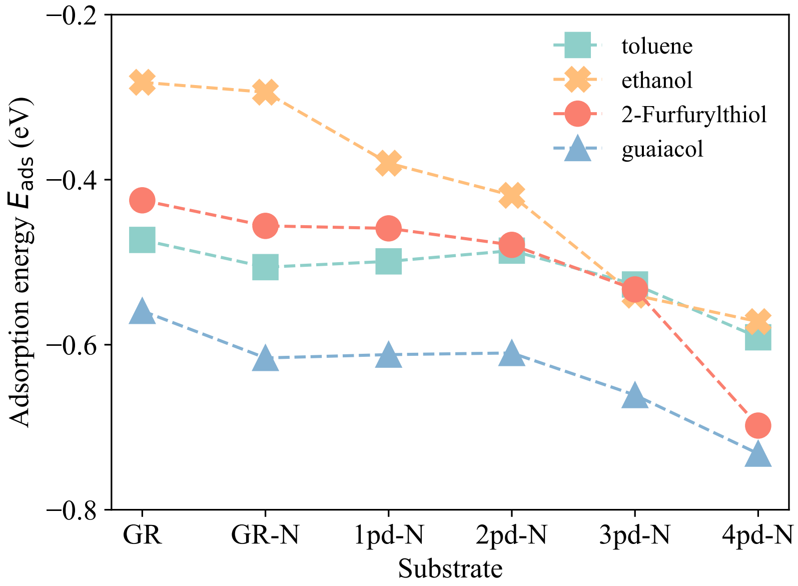

Figure S5: Adsorption energy comparison for the odorants adsorption on different substrates is shown.

S4 Adsorption energy

The strength of interaction between substrate and adsorbate is described by the adsorption energy E_{ads}, which is defined as:

$$E_{\mathrm{ads}} = E_{\mathrm{cplx}} - E_{\mathrm{s}} - E_{\mathrm{a}}, \tag{S1}$$

where E_{cplx} is the total energy of the optimized adsorbate-substrate complex system, while E_{s} and E_{a} are the total energies of the isolated substrate and gas molecule, respectively. The lower the adsorption energy is, the more stable the adsorption configuration is. The adsorption energies for each adsorption system are illustrated in Figure S5. Generally, the guaiacol system exhibits the lowest adsorption energy on all substrates. This can be attributed to the robust van der Waals interactions facilitated by π-π stacking interactions, coupled with the strong electrostatic forces originating from its two oxygen atoms. In contrast, ethanol displays weaker interactions due to its smaller molecular mass. Toluene, despite its lower molecular weight, exhibits higher adsorption energy, primarily due to π-π stacking interactions, similar to guaiacol. 2-Furfurylthiol shares a similar molecular mass with guaiacol, however, the S atom situated between the N-doped graphene substrate seems to have a repulsive interaction with the substrate, resulting in weakened interactions, as the red dots are overall above the blue triangles indicating the increase of the adsorption energy. Another noteworthy observation from the graph is the descending trend in E_{ads} from pristine graphene (GR) to N-doped graphene (GR-N) and to multiple-pd-N substrates. Specifically, a substantial drop in E_{ads} is observed when transitioning from 2pd-N to 3pd-N and 4pd-N substrates. Consequently, it can be inferred that the introduction of nitrogen into graphene enhances the adsorption of these VOC molecules.
